# Time to Optimize Supplementation: Modifying Factors Influencing the Individual Responses to Extracellular Buffering Agents

**DOI:** 10.3389/fnut.2018.00035

**Published:** 2018-05-08

**Authors:** André B. Heibel, Pedro H. L. Perim, Luana F. Oliveira, Lars R. McNaughton, Bryan Saunders

**Affiliations:** ^1^Applied Physiology and Nutrition Research Group, University of São Paulo, São Paulo, Brazil; ^2^Laboratory of Nutritional Biochemistry, University of Brasília, Brasília, Brazil; ^3^São Camilo University Centre, São Paulo, Brazil; ^4^School of Physical Education and Sport, University of São Paulo, São Paulo, Brazil; ^5^Sports Nutrition and Performance Group, Department of Sport and Physical Activity, Edge Hill University, Ormskirk, United Kingdom; ^6^Department of Sport and Movement Studies, Faculty of Health Science, University of Johannesburg, Johannesburg, South Africa; ^7^Rheumatology Division, Faculty of Medicine, University of São Paulo, São Paulo, Brazil; ^8^Institute of Orthopaedics and Traumatology, Faculty of Medicine, University of São Paulo, São Paulo, Brazil

**Keywords:** buffering agents, alkalosis, bicarbonate, citrate, lactate

## Abstract

Blood alkalosis, as indicated by an increased blood bicarbonate concentration and pH, has been shown to be beneficial for exercise performance. Sodium bicarbonate, sodium citrate, and sodium or calcium lactate, can all result in increased circulating bicarbonate and have all independently been shown to improve exercise capacity and performance under various circumstances. Although there is considerable evidence demonstrating the efficacy of these supplements in several sports-specific situations, it is commonly acknowledged that their efficacy is equivocal, due to contrasting evidence. Herein, we discuss the physiological and environmental factors that may modify the effectiveness of these supplements including, (i) absolute changes in circulating bicarbonate; (ii) supplement timing, (iii) the exercise task performed, (iv) monocarboxylate transporter (MCT) activity; (v) training status, and (vi) associated side-effects. The aim of this narrative review is to highlight the factors which may modify the response to these supplements, so that individuals can use this information to attempt to optimize supplementation and allow the greatest possibility of an ergogenic effect.

## Introduction

Substances capable of increasing extracellular buffering capacity to combat exercise induced acidosis have been researched for almost a century. Sodium bicarbonate, sodium citrate, and sodium/calcium lactate can all result in alkalosis, indicated by an increase in blood pH and circulating bicarbonate, and all have independently been shown to improve exercise capacity and performance under various circumstances (1). Despite considerable evidence demonstrating these supplements to be effective in specific situations (2, 3), it is commonly acknowledged that their efficacy is equivocal due to contrasting evidence. Contradictory results from different investigations are commonly cited to highlight that the ergogenic effect from increased bicarbonate concentration is highly variable and is due to a variability in the individual responses. However, this may be oversimplifying the complex nature of individual responses (i.e., physiological and genetic differences), whilst also overlooking experimental differences (loading and exercise protocols), sample populations (i.e., untrained; physically active; trained individuals), and design flaws (e.g., exercise unlikely to be affected by increased buffering capacity).

Recent developments in the way in which researchers analyze and interpret data is moving away from group means and toward more in-depth analysis of individuals responses (4). This is because we now know that there are several contributing factors that determine how individuals respond to supplementation, including their genetic composition, training status, habitual diet, and other circumstantial factors. Identification of any modifying factors that may alter the individual response to supplementation with extracellular buffering agents would provide vital information to clinicians, coaches, and athletes about the likelihood of gaining a positive or negative response. This would aid them in making fully informed decisions and optimize each individual's personal supplementation protocol. The aim of this review is to summarize the current evidence on the potential modifying factors underlying the individual response to supplementation with extracellular buffering agents.

## The role of pH in skeletal muscle fatigue

The ability of skeletal muscle tissue to maintain energy production and generate mechanical work is closely linked to performance in a variety of sports. The high turnover rate of skeletal muscle ATP that is seen during high-intensity exercise increases hydrogen ion (H^+^) production, leading to muscle acidosis that is associated with performance loss (5). This is because an exercise-induced metabolic acidosis, characterized by a higher H^+^ production rather than removal rate, can decrease energy substrate generation via glycolytic pathways by reducing the activity of key enzymes such as glycogen phosphorylase and phosphofructokinase (6). The H^+^ also compete with calcium ions for the troponin binding site, directly hindering the muscle's contraction capacity (7). Oxidative phosphorylation can also be inhibited by acidosis (6) while resynthesis of phosphorylcreatine may also be compromised at low pH (8). Thus, a myoplasmic drop in pH drives an inability to produce the desired or required power output with a subsequent loss of exercise performance (9), although not all agree (10).

The body has several endogenous systems to control pH homeostasis; this balance is maintained by intracellular and extracellular buffers which can accept or release H^+^ to prevent dramatic pH changes. In muscle, intracellular physicochemical buffers such as organic and inorganic phosphates, bicarbonate anions, and histidine containing dipeptides, are the primary mediators of pH homeostasis. There is also an active and passive transport of H^+^ out of the muscle into the blood mediated by transport systems; during intense activity, H^+^ efflux is mainly mediated by the lactate-proton transporters, namely monocarboxylate transporter 1 (MCT1) and 4 (MCT4) (11). Thereafter, the H^+^ are buffered by the circulating anion bicarbonate (${\text{HCO}}_{3}^{-}$), which forms carbonic acid, a weak acid. These endogenous buffering systems are well-regulated and highly efficient under normal physiological conditions (12). However, these systems can be quickly overwhelmed by the accumulation of H^+^ during exercise, particularly when the intensity is high. Thus, increasing the buffering contribution of one or more of these systems is a feasible way to improve control of systemic pH changes and maintain exercise performance.

## Extracellular buffering agents

Several buffering agents are employed as ergogenic supplements, including sodium bicarbonate (SB), sodium citrate (SC), sodium lactate (SL), and calcium lactate (CL); the independent mechanisms through which they increase circulating bicarbonate will not be discussed here (for review see Lancha Junior et al. (1)). Nonetheless, all substances are ingested with the same focus: to increase the extracellular concentration of bicarbonate, increasing H^+^ efflux out of the working muscle, thereby contributing to muscle acid-base balance during exercise, which may lead to an improved performance.

The ability of increased circulating bicarbonate to improve exercise capacity and performance has been extensively studied. It is widely acknowledged that the most effective extracellular buffer is SB, with numerous narrative reviews (13, 14) and meta-analyses (2, 3, 15, 16) demonstrating its efficacy. Meta-analytic data have suggested SC supplementation to be ineffective for performance (2), while limited data on SL and CL mean that no meta-analytical data on their efficacy currently exists. Despite this, supplementation with SC, SL, and CL has been shown to improve exercise capacity and performance on numerous independent occasions. Contrasting results in study outcomes following ingestion of these supplements is often cited as evidence that the response to these buffering supplements is variable. Such generalized statements do not consider the numerous contributing and modifying factors that influence the response to supplementation and do not allow any single individual to identify whether they are likely to benefit from supplementation or not. Thus, it is of importance to determine the factors that may contribute to an individual's response to these supplements.

## Potential influencing factors on the efficacy of extracellular buffers to improve exercise capacity and performance

### Increases in circulating bicarbonate

To gain a competitive advantage from increased extracellular buffering capacity, there needs to be an increase in circulating bicarbonate following supplementation. Theoretically, any increase in bicarbonate would lead to a corresponding increase in buffering capacity; what the minimal increase necessary is to elicit performance gains is currently unknown. Carr et al. (17) have suggested that a +5 mmol·L^−1^ increase from baseline levels is required to have a potential ergogenic benefit in exercise performance, while a +6 mmol·L^−1^ increase leads to almost certain ergogenic benefits. Despite this, no study to date has directly investigated the minimal increase necessary for performance gains or linked individual increases to change in performance. Saunders et al. (18) determined whether there were any correlations between blood values (bicarbonate, pH, base excess) and exercise capacity. Surprisingly, we did not show any relationship between the magnitude of change in circulating bicarbonate (or any other measure) and subsequent changes in exercise capacity.

Jones et al. (19) showed that no individual's maximal increases in circulating bicarbonate with a 0.1 g·kg^−1^ BM dose reached a +5 mmol·L^−1^ increase, which could explain why this dose appears to be ineffective for exercise performance (20). A 0.2 g·kg^−1^ BM dose, however, was effective at reaching this threshold in all individuals (19), although the mean increase 60 min post-ingestion was < +5 mmol·L^−1^. Nonetheless, it is possible that 60 min post-ingestion could coincide with a sufficient increase (i.e., > +5 mmol·L^−1^) in bicarbonate for some individuals. This could partially explain why a 0.2 g·kg^−1^ dose appears to be effective in some (20, 21), but not all (22–24), studies when exercise was performed 1 h post-supplementation. The time to reach this suggested minimal threshold varies between individuals (19), meaning it is possible that a higher proportion of individuals had not attained sufficient alkalosis in the studies showing no effect than those showing an effect, contributing to the group effects shown.

**Figure 1:**
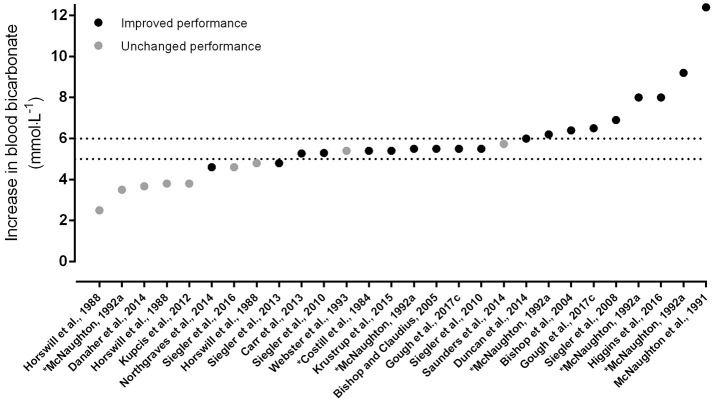
Increases in blood bicarbonate from baseline following acute supplementation with sodium bicarbonate, in order of magnitude of change. Data points indicate whether exercise performance was improved with supplementation (dark circles) or not (light circles). The dotted lines indicate the thresholds for the zone of a potential ergogenic effect (+5 mmol·L^−1^) and the zone of an almost certain ergogenic effect (+6 mmol·L^−1^); taken from Carr et al. (2) and Jones et al. (19). ^*^Denotes data estimated from graphs using specialized software (18, 20, 21, 23, 26, 29, 30, 33, 38, 42–53).

A 0.3 g·kg^−1^ BM dose of SB is the most commonly employed in the literature and appears to stem from the work of McNaughton who showed this to be most effective compared to lower and higher doses (20). Mean increases in circulating bicarbonate prior to exercise following a 0.3 g·kg^−1^ BM dose of SB is approximately +5–6 mmol·L^−1^ (25–27), and thus should be sufficient to improve exercise capacity and performance (19). Indeed, numerous studies have shown exercise gains with SB at this dose (28–38), though similarly, substantial data exist showing no effect following supplementation (26, 27, 34, 39–43). This may, in part, be explained by the absolute increases in blood bicarbonate shown prior to exercise, which appears to differ substantially between studies (Figure 1). Certainly, this visual demonstrates the importance in mean increases in circulating bicarbonate prior to subsequent exercise performance, although it must be acknowledged that several further contributing factors may account for this variation including exercise models not limited by H^+^ accumulation, genotype, associated-side effects, and individual variation in the response to supplementation.

Few studies have investigated the effect of repeated supplementation in the same individuals using the same exercise protocol. We previously showed that SB supplementation in the same individual produced consistent blood bicarbonate responses, but this only translated into a mean improvement in one of the four sessions when SB was ingested (27). Interestingly, only one individual improved on all four occasions and nine others on at least one occasion, suggesting that subsequent exercise performance is more variable than blood responses. Nonetheless, although mean increases in circulating bicarbonate were approximately +6 mmol·L^−1^ during each session, not all individuals may have achieved a sufficient increase in circulating bicarbonate on every occasion. Additionally, inconsistencies may also be due to the training status of the participants who were recreationally active; trained individuals are likely to perform more consistently with lower variation between tests and thus may be better able to consistently take advantage of increased buffering capacity. Indeed, Carr et al. (41) showed 2,000 m rowing performance was reliable following repeated acute (0.3 g·kg^−1^ BM) and chronic (0.5 g·kg^−1^·day^−1^ BM for 3 days) supplementation in well-trained rowers, although performance was not improved. Trained cyclists and triathletes did consistently improve their cycling tolerance when administered SB on repeated occasions (35). Thus, it is possible that a proportion of the inconsistency in the response to SB (and potentially SC, CL, and SL) may be attributed to the training status of the individual and it would be of interest to determine the within-participant repeatability following supplementation using trained individuals.

**Figure 2:**
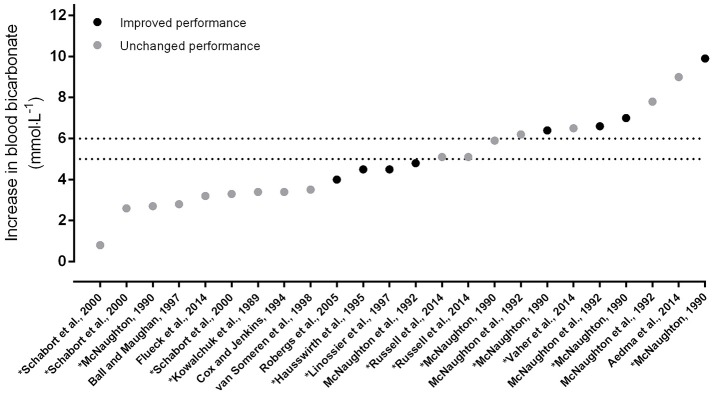
Increases in blood bicarbonate from baseline following acute supplementation with sodium citrate, in order of magnitude of change. Data points indicate whether exercise performance was improved with supplementation (dark circles) or not (light circles). The dotted lines indicate the thresholds for the zone of a potential ergogenic effect (+5 mmol·L^−1^) and the zone of an almost certain ergogenic effect (+6 mmol·L^−1^); taken from Carr et al. (2) and Jones et al. (19). ^*^Denotes data estimated from graphs using specialized software (24), (54–66).

Like the 0.3 g·kg^−1^ BM dose of SB, the most commonly employed dose of SC is 0.5 g·kg^−1^ BM and can be attributed to the pioneering work of McNaughton (54). Significant increases in bicarbonate were shown 90 min following a dose as low as 0.1 g·kg^−1^ BM, but since circulating bicarbonate increased in a linear fashion, and the greatest amount of work completed during a 60-s maximal exercise test was following a 0.5 g·kg^−1^ BM dose, this was considered the most effective. And yet, despite impressive increases in blood bicarbonate following SC supplementation, numerous studies have shown no effect on exercise capacity or performance (55–57). In fact, meta-analytic data showed an unclear effect of SC on exercise, with pre-exercise alkalosis associated with a small but unclear effect on performance (2). Indeed, despite clear increases in blood bicarbonate, several studies have shown no positive effect on subsequent exercise capacity or performance theoretically limited by acidosis (Figure 2). Aside from other confounding factors, discussed herein that may have modified the ergogenic effect of supplementation with SC, these increases may be counteracted by some other physiological response which inhibits the ergogenic effect of increased buffering capacity.

**Figure 3:**
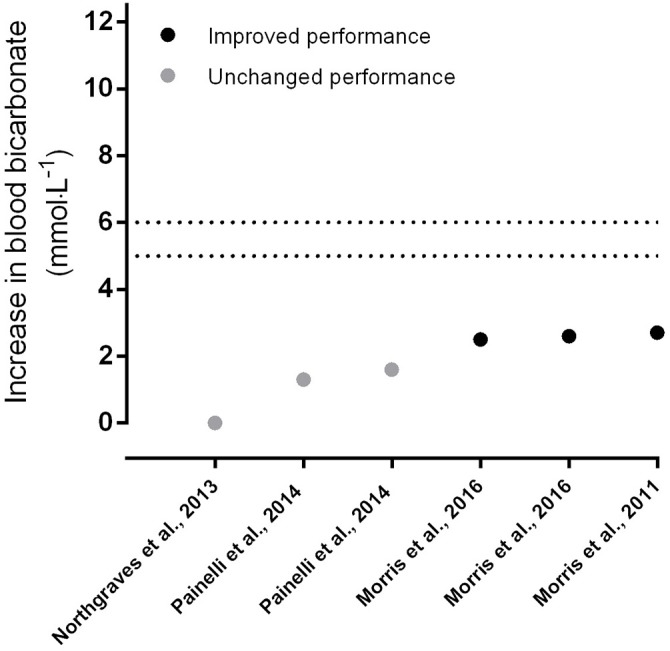
Increases in blood bicarbonate from baseline following acute supplementation with calcium or sodium lactate, in order of magnitude of change. Data points indicate whether exercise performance was improved with supplementation (dark circles) or not (light circles). The dotted lines indicate the thresholds for the zone of a potential ergogenic effect (+5 mmol·L^−1^) and the zone of an almost certain ergogenic effect (+6 mmol·L^−1^); taken from Carr et al. (2) and Jones et al. (19). ^*^Denotes data estimated from graphs using specialized software (26, 68, 70, 72).

Increases in blood bicarbonate following ingestion of calcium or sodium lactate appear less obvious. Several studies have shown increases of the magnitude of ~+3 mmol·L^−1^ (67, 68) while further studies showed no increase in circulating bicarbonate following acute (26) and chronic (69) supplementation, which explains the lack of an ergogenic effect in these studies. The +3 mmol·L^−1^ increase shown by Morris et al. (68) was sufficient to improve exercise tolerance by 17% during a cycling capacity test. Similarly, Morris et al. (70) showed improved high-intensity exercise capacity with mean bicarbonate increases of only +2.5 and +2.6 mmol·L^−1^ following supplementation of 120 and 300 mg·kg^−1^ BM of CL. Lactate supplementation improved running performance by a modest 1.7% (71), although changes in bicarbonate in this study cannot be determined since blood was only sampled at a solitary timepoint immediately prior to exercise. Low (150 mg·kg^−1^ BM) and high (300 mg·kg^−1^ BM) doses of CL lead to only moderate increases of +2 mmol·L^−1^ of bicarbonate, with no subsequent effect on repeated-bout high-intensity exercise (72). Certainly, the modest increases in blood bicarbonate with lactate supplementation appear to be the primary factor behind the lack of an ergogenic effect shown in some, but not all, studies with lactate supplementation (Figure 3). Certainly, the individual range of bicarbonate increases could have contributed to this variability as, for example, the range in increases with low and high doses of calcium was between +0.4 and +6.7 mmol·L^−1^ and changes in bicarbonate were positively associated with the improvements in exercise capacity (70). More studies should relate the individual increases in circulating bicarbonate to changes in performance to provide insight into this relationship.

It is currently unclear what the minimal increase in circulating bicarbonate necessary is to result in an improvement in exercise performance. This has been suggested to be of the order of +5 mmol·L^−1^ (17), although studies have shown mean increases as little as +2.5 mmol·L^−1^ are sufficient to improve exercise tolerance (70), while +2 mmol·L^−1^ generally do not (72). Further research should investigate the smallest worthwhile change in blood bicarbonate that can consistently lead to exercise gains.

### Ingestion timing

Siegler et al. (25) showed that increases in circulating bicarbonate and absolute concentration are similar at 60, 120, and 180 min post-ingestion of 0.3 g·kg^−1^ BM SB. Since performance during their repeated sprint protocol was not different when performed at these different post-ingestion timepoints, it could be assumed that precise timing of supplementation is of little importance to obtain performance gains. Nonetheless, recent interest has focused on the peak changes in circulating bicarbonate, with theory suggesting that maximal increases in blood bicarbonate will lead to maximal performance benefits, or at least the greatest chance of improvements. Most studies have employed a standardized supplementation protocol at a uniform timepoint. Although the data of Siegler et al. (25) showed that mean changes in circulating bicarbonate did not differ between timepoints, it is unlikely that all individuals performed exercise at a point which coincided with peak alkalosis.

A recent spate of investigations reporting the time course changes in blood markers following acute supplementation with SB (19, 73, 74), SC (75), and lactate (26, 72) suggest that a uniform supplementation timepoint is unlikely to be optimal for all individuals. The overwhelming conclusion that can be drawn from these independent investigations is that the time-to-peak bicarbonate concentration differs drastically between individuals, as does the absolute maximal change. Jones et al. (19) showed that peak bicarbonate concentration occurred between 30 and 150, 40 and 165, and 75 and 180 min following 0.1, 0.2, and 0.3 g·kg^−1^ BM of SB. Another study showed one individual who peaked only 10 min following supplementation of 0.3 g·kg^−1^ BM of SB (76). Absolute maximal changes in bicarbonate following SB ingestion varies greatly between individuals (Table 1), which could be a contributing factor to why some individuals respond more than others. Urwin et al. (75) showed that peak bicarbonate occurred much later than the commonly employed time given for supplementation with SC; indeed, it has recently been suggested that this may be a key factor contributing to the unclear effect shown with SC (77). Thus, if the greatest likelihood of an ergogenic effect occurs at the moment of peak circulating bicarbonate, then it is of great importance to determine individual time-to-peak bicarbonate following supplementation.

**Table 1:** Minimum (Min), maximum (Max), and mean time-to-peak and absolute maximal increases in blood bicarbonate concentration following supplementation with 0.1, 0.2, and 0.3 g·kg^−1^ BM sodium bicarbonate (SB) in Jones et al. (19).

**Dose**	**Blood bicarbonate**					
**SB (g·kg^−1^ BM)**	**Time-to-peak (min)**			**Absolute change (mmol·L^−1^)**		
	**Min**	**Max**	**Mean**	**Min**	**Max**	**Mean**
0.1	30	150	78 ± 34^*^^Δ^	+2.0	+5.0	+3.6 ± 0.8^*^
0.2	40	165	98 ± 32^*^	+5.1	+8.1	+6.1 ± 0.9^*^
0.3	75	180	123 ± 36	+6.0	+12.3	+8.2 ± 1.4

* P < 0.05 from 0.3 g·kg^−1^BM;

Δ *P < 0.05 from 0.2 g·kg^−1^ BM*.

Determination of the time-to-peak bicarbonate concentration is only worthwhile for repeat ingestion if subsequent blood responses are reproducible. The consistency in blood responses following supplementation with extracellular buffers has only been determined with SB. Although both blood pH and bicarbonate responses showed good reliability following repeated supplementation with both 0.2 and 0.3 g·kg^−1^ BM doses of SB, blood bicarbonate responses were more reproducible, suggesting any individualized ingestion strategy should be based upon the time-to-peak bicarbonate (73). Nonetheless, although no statistical differences were shown in the time-to-peak, or the absolute maximal changes, it currently remains unclear whether minor changes in circulating bicarbonate significantly influence changes in exercise performance.

Two studies have investigated the effect of peak circulating bicarbonate concentration on exercise performance, both following SB supplementation. Overall repeated sprint performance was significantly improved with 0.3 g·kg^−1^ BM of SB compared to placebo and control (76). More recently it has been shown that 4-km cycling time-trial performance could be improved following both 0.2 and 0.3 g·kg^−1^ BM of SB when individualized to the time-to-peak blood bicarbonate (48). These findings are interesting considering two previous investigations showed no effect of SB supplementation on 4-km time-trial performance following a 0.3 g·kg^−1^ BM dose. Discrepancies could be due to timing and subsequent increases in circulating bicarbonate. Mean bicarbonate increases in Gough et al. (48) were +6.5 ± 1.3 mmol·L^−1^, which is higher than the approximate +3 mmol·L^−1^ (78) and +5 mmol·L^−1^ (79) shown 150 and 100 min following supplement ingestion in the other studies. Despite these initial positive results, no data exist directly comparing exercise performed at the moment of peak circulating bicarbonate to a standardized time (e.g., 60 min) following supplementation in the same participants. As it stands, it cannot currently be concluded that exercise performed at peak bicarbonate concentrations elicits greater exercise improvements than normally seen with standardized ingestion prior to exercise.

Interestingly, recent data with SB show that determining time-to-peak blood bicarbonate concentration may allow a lower dose to be employed since maximal increases with 0.2 g·kg^−1^ BM were similar to those seen with 0.3 g·kg^−1^ BM, while 4-km time-trial performance was not different between these two doses (80). The absolute difference in maximal bicarbonate increases between these doses was ~1 mmol·L^−1^, suggesting that such a difference may not be meaningful. Since peak bicarbonate is only assessed by the absolute maximal value, and not via some statistical inference, it is possible that minor fluctuations in the actual bicarbonate concentration following large increases may not significantly influence subsequent exercise further, although work remains to be done to determine the smallest worthwhile change in blood bicarbonate that would influence exercise performance.

### Exercise task

An increase in buffering capacity via increases in circulating bicarbonate can lead to improvements in exercise capacity and performance, and the magnitude of this increase in buffering capacity appears to influence the likelihood of an effect, although even following substantial increases not all research is unanimous. These equivocal findings may also be due to the exercise protocols employed. If improvements are to be gained from increases in buffering capacity, exercise must be limited by increases in H^+^ accumulation. Certainly, it has been suggested that short-duration exercise (in this case a 30 s cycle sprint) is unaffected by changes in muscle pH (81), while more endurance based activities rely increasingly on aerobic energy sources with no further increases in muscle acidosis. Therefore, improvements following supplementation with SB, SC, and CL and SL, are likely confined to specific exercise tasks which are significantly influenced by changes in acid-base balance. In fact, beta-alanine supplementation, which increases intracellular buffering capacity via increased muscle carnosine content, has been shown to be most effective during high-intensity exercise of 30 s to 10 min in duration, while longer and shorter duration exercise was unaffected (82). Since the mechanism of action of increased circulating bicarbonate is like that of beta-alanine supplementation (i.e., increased buffering capacity), comparable results would be expected.

Indeed, research appears to suggest exactly this. Despite similar increases in circulating bicarbonate following a 0.3 g·kg^−1^ BM dose of SB, exercise lasting 10 and 30 s in duration was unaffected, while exercise 120 and 240 s in duration was significantly improved (83). Identical results were shown following supplementation with 0.5 g·kg^−1^ BM of SC (63). Although some evidence suggests that buffering agents may be of benefit to longer duration exercise (36, 84), most studies report no improvements in continuous endurance exercise following supplementation (26, 57, 58, 85, 86). Multiple-bout high-intensity exercise, which has been shown to result in greater muscle acidosis than continuous supra-maximal exercise (87, 88), appears particularly susceptible to improvements with these supplements (21, 28–30, 38, 76, 89). Interestingly, Higgins et al. (34) showed that SB improved exercise capacity at 100% peak mean minute power, but not at 110 or 120%, despite the duration of all these exercise tasks being within the timeframe where an effect may be most likely expected (82).

Taken collectively, these results suggest that the likelihood of gaining a competitive edge from increased bicarbonate may be, in part, dependent on the exercise performed; the duration and intensity of the exercise appear to be key factors in this. Thus, the exercise task performed will be a determining factor in whether supplementation will be beneficial and cannot be overlooked; exercise gains with these substances should only be expected during exercise limited by the accumulation of H^+^. Individuals should base their decision to supplement on their own exercise demands and the likelihood of gaining a worthwhile improvement therein. These are likely to be continuous high-intensity exercise tasks, such as 4 km cycling, 100 and 200 m swimming, and 2,000 m rowing, or repeated high-intensity activities such as those performed during team sports (e.g., football, hockey, basketball, etc).

### Hydrogen ion transporters

The H^+^ that accumulate in muscle throughout high-intensity exercise are predominantly removed by MCT1 and MCT4, through co-transport with lactate in a 1:1 ratio (11, 90). The sodium/hydrogen transport system (NHE) may also contribute to this process (91), although its relative contribution during exercise has been suggested to be minimal (90, 92). It is widely acknowledged that increased blood bicarbonate increases the activity of these MCT transporters, increasing the efflux of H^+^ out of the muscle and reducing muscle acidosis (93). Surprisingly, no study to date has directly measured the effect of increased circulating bicarbonate via supplementation on the activity of these transporters in humans. It is currently unclear whether the relationship between the increased bicarbonate and the activity of these transporters is intrinsically associated.

Lactate transport has been shown to be elevated in athletes (94). This makes sense since training interventions have been shown to increase the abundance and activity of MCT transporters (95, 96). Furthermore, lactate transport capacity (and subsequently H^+^ transport) was related to the occurrence of type I muscle fibers (97). MCT1 and MCT4 are both expressed in human skeletal muscle, although MCT1 is more prevalent in type I fibers and MCT4 in type II fibers (98). The higher affinity of MCT4 (Km = 28–34 mM) compared to MCT1 (Km = 4–6 mM) for lactate, in addition to its higher prevalence in glycolytic fibers, suggests that MCT4 may be more important for efflux out of the working muscle (99). Hence, it could be suggested that any differences in training status or muscle fiber type predominance (i.e., trained sprinters vs. untrained individuals) may modify the response to supplementation with extracellular buffering agents. Indeed, this could, in part, explain some of the variation in responses between trained and non-trained individuals; a meta-analysis showed that the effect of SB supplementation on exercise was significantly lower for specifically trained compared to recreationally trained individuals (16). However, the exact mechanisms for this reduced effect has not been investigated and thus, suggestion that this may be due to differences in MCT transporter activity is currently highly speculative and warrants further investigation.

Polymorphisms in the MCT transporters may also influence an individual's response to supplementation. A single-nucleotide polymorphism in the gene coding for MCT1, for example, can influence the lactate/H^+^ co-transport across the sarcolemma (100). The T allele carriers have been shown to have a reduced MCT1 lactate transport (101, 102), TT homozygotes reach the ventilatory threshold at higher speeds (103), while T allele frequency has been shown to differ between athletes of different modalities (100, 104). Despite this, the effect of this polymorphism on high-intensity exercise performance, with or without supplementation, is currently unclear. Although genetic variations of the MCT4 gene exist (105), to our knowledge, no study has investigated the relevance of any polymorphisms on lactate/H^+^ flux and its effect on exercise performance. It is apparent that polymorphisms in the genes encoding for MCT1 and MCT4 could alter their activity during high-intensity activity; it could thus be implied that genetic differences may also modify the individual response to supplementation with buffering agents, although no data currently exist. It is essential that more research on genotypic differences in MCT transporters and response to supplementation is undertaken before any recommendations can be made.

### Associated side-effects

An evident moderator of the efficacy of buffering agents to improve exercise capacity and performance, is their associated side-effects. The occurrence of gastrointestinal (GI) discomfort with these supplements is common, with stomach cramps, nausea, vomiting, and diarrhea among some of the most frequent complaints with SB and SC (17, 75), although the only reported side-effects with lactate supplementation was increased incidence of belching and flatulence (72). This has obvious implications for athletes considering supplementing during competition and is likely a contributing factor to why incidence of supplementation with such agents is low (106).

Following ingestion of SB, it is dissociated in the stomach acid to form sodium (Na^+^) and bicarbonate, much of the latter of which is swiftly neutralized by H^+^, thereby producing carbon dioxide [CO_2_] (107). The production of CO_2_ in the stomach may cause gastric discomfort with symptoms including bloating and abdominal pain; nausea and vomiting are other commonly reported side effects (17). A contributing factor to the intensity of any side-effects with SB is certainly dose, as McNaughton et al. (20) reported increasing discomfort with increasing doses above 0.3 g·kg^−1^ BM, with no concomitant increases in performance. The typical 0.3 g·kg^−1^ BM often results in reported side-effects which may moderate performance (2, 16), although whether they directly influence performance may be disputed. Indeed, Price and Simons (40) reported no association between GI discomfort and performance while data from our laboratory has shown on several occasions that exercise capacity may be modified by GI discomfort (18, 27). Specifically, all individuals reporting discomfort worsened performance, and reanalyzing data following the removal of these individuals modified the group level statistics, turning a non-significant result into a significant result. Thus, it is apparent that minimizing the discomfort associated with SB supplementation could increase the likelihood of a positive response, both at the individual and group level. A potential solution is to ingest SB in gastro-resistant capsules, avoiding neutralization in the stomach and the associated side-effects; this theory is currently being tested in our laboratory.

The timing of supplement ingestion may be a key modifiable component that can increase or decrease the likelihood of an ergogenic effect not only due to increased circulating bicarbonate, but also due to the associated side-effects. Siegler et al. (25) reported lower symptoms of GI discomfort 180 min following SB supplementation (compared to 60 and 120 min). The most intense symptoms with SC were 60–120 min following supplementation (75). Since supplements are commonly ingested at a standardized time, often 60 min prior to the commencement of exercise as in the case of SB, this suggests that most studies may have forced individuals to exercise at a time when they experience sufficient discomfort to negatively impact their performance. Thus, adopting an individual time-to-peak bicarbonate supplementation protocol would likely avoid performing exercise when associated side-effects are most intense. Although we have shown that side-effects with these supplements could modify their ergogenic effect, it cannot explain the lack of an effect in all individuals. Nonetheless, it is apparent that offsetting any discomfort with supplementation will avoid any possibility that side-effects will affect the exercise response.

## Optimizing supplementation

It is becoming apparent that timing of ingestion, may be one of the key moderating factors to the ergogenic response to increased circulating bicarbonate. Coinciding exercise with the point at which bicarbonate peaks is likely to maximize the chance of exercise improvements since buffering capacity will be maximized. Importantly, early reports suggest that the time at which peak bicarbonate occurs with SB is repeatable within individuals (73); more information is needed regarding consistency in blood bicarbonate responses to other buffering agents (SC, SL, and CL). Furthermore, peak GI discomfort has been shown to occur earlier than maximal increases in blood bicarbonate following SB (73, 80) and SC (75) supplementation. Reported side-effects with lactate supplementation are minimal and mean peak bicarbonate concentration occurred after 90 min although further information is necessary regarding the individual time course response. Altogether, data suggest that individualizing supplementation timing with these agents may provide the best opportunity of gaining an ergogenic effect by coinciding the moment of exercise with peak bicarbonate concentration following the selected dose.

In fact, supplement dose is another highly modifiable factor that could be adapted to optimize supplementation. Increasing doses of SB above 0.3 g·kg^−1^ BM results in increased incidence of GI discomfort, while 0.1 g·kg^−1^ BM appears insufficient to result in exercise improvements (20). Although 0.3 g·kg^−1^ BM has long been considered the optimal dose, recent evidence suggests that 0.2 g·kg^−1^ BM may be equally efficient at improving performance (80). Considering the lower incidence of GI discomfort with similar gains in bicarbonate, a 0.2 g·kg^−1^ BM ingested at a timepoint specific to the individual may be the most effective way of maximizing the chances of an ergogenic effect with SB. SC increased blood bicarbonate in a linear manner in doses increasing from 0.1 to 0.5 g·kg^−1^ BM (54); doses above 0.5 g·kg^−1^ BM of SC showed no further gains in blood bicarbonate but increased the incidence and severity of side-effects (75) suggesting 0.5 g·kg^−1^ BM of SC to be the optimal dose. Lactate supplementation results in modest increases in bicarbonate regardless of a 0.15 or a 0.3 g·kg^−1^ BM dose although no significant GI discomfort. Nonetheless, current doses of up to 0.3 g·kg^−1^ BM with CL or SL may be insufficient to improve exercise though further work is necessary to ascertain whether higher doses may be more beneficial.

## Practical recommendations

Individuals aiming to supplement with these substances should trial them outside of competition to see what works for them and adapt and optimize their individual supplementation strategies according to their own personal needs and responses. Since the time course response has been shown to be consistent (at least following SB ingestion), ingestion should occur at a time that means exercise begins when bicarbonate concentration has increased above this +5–6 mmol·L^−1^ threshold. Although there may be logistical difficulties, it would be necessary to determine the timeframe during which individuals increase their bicarbonate levels above +5–6 mmol·L^−1^, and side-effects are minimal, so that they can adapt their supplementation timing accordingly to optimize their chances of an effect. Individuals should be wary that any improvements in exercise are likely to be marginal, although this may be worthwhile for highly-trained or elite athletes. Furthermore, the type of exercise that individuals undertake will influence the likelihood of any benefit from increased bicarbonate. Short-duration high-intensity exercise, such as 4 km cycling, 2,000 m rowing, and 200 m swimming, are examples of activities likely to profit from increased buffering capacity and there is also evidence that individuals involved in prolonged intermittent exercise may benefit from supplementation. It appears the likelihood of obtaining an ergogenic effect is greater when SB is ingested compared to SC, SL, or CL, and it is the recommendation of these authors that 0.2–0.3 g·kg^−1^ BM SB is taken as the preferred supplement where possible to improve high-intensity exercise capacity and performance limited by acidosis.

## Conclusions and future direction

**Figure 4:**
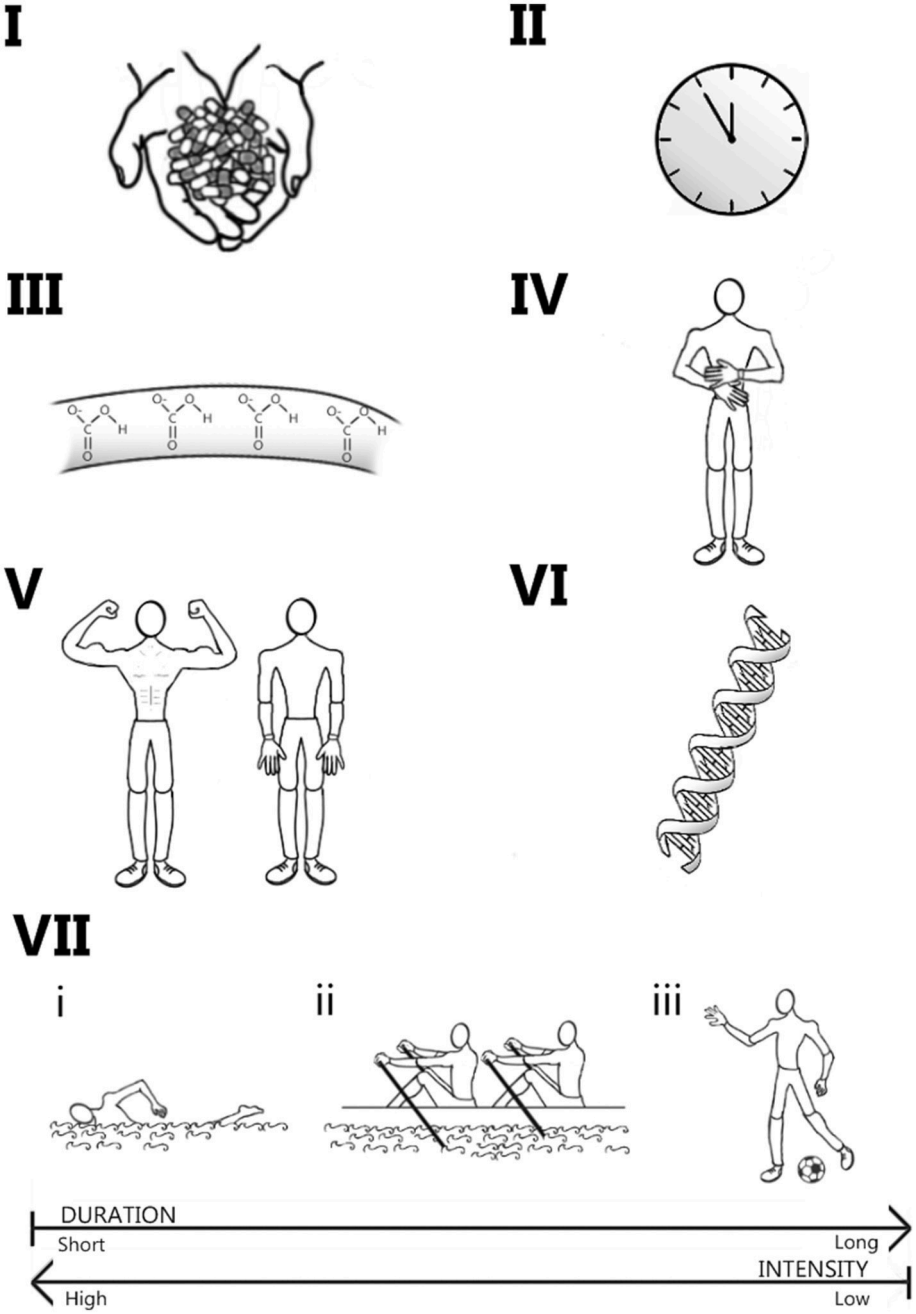
Overview of the factors which may modify the response to supplementation with sodium bicarbonate, sodium citrate, and calcium and sodium lactate. The dose (Panel I) and timing of ingestion (Panel II) can influence both the increases in circulating bicarbonate (Panel III) and associated side-effects (Panel IV) at the moment of exercise, impacting upon the likelihood of a performance benefit. Training status (Panel V) and genetic make-up (Panel VI) of the individual may further modify this response. The chances of a positive response may also be modified by the duration and intensity of the exercise task being performed (Panel VII): short-duration continuous exercise, such as 100 and 200 m swimming (i) and 2,000 m rowing (ii), and prolonged intermittent activity, such as football (iii), may be particularly susceptible to improvements with these supplements.

Sodium bicarbonate, sodium citrate and sodium/calcium lactate are supplements to increase circulating bicarbonate and have all independently been shown to improve exercise capacity and performance under various conditions. However, several factors may modify their ergogenic effects including supplement timing and dose, absolute changes in circulating bicarbonate, the exercise task being performed, MCT activity, training status, and associated side-effects (Figure 4). Emerging evidence suggests that, to maximize the chances of an ergogenic effect, supplementation should occur at a time point that results in exercise being performed at the moment of peak bicarbonate concentration, with minimal or no side-effects. More information is required regarding each supplement independently and their interaction with the individual, genotype, and the environment; these have been detailed throughout this article.

## Author contributions

BS is responsible for the conception of the work. AH, PP, LO, and BS are responsible for the writing of the manuscript. LM reviewed the manuscript.

### Conflict of interest statement

The authors declare that the research was conducted in the absence of any commercial or financial relationships that could be construed as a potential conflict of interest.
